# The importance of uncertainty quantification in model reproducibility

**DOI:** 10.1098/rsta.2020.0071

**Published:** 2021-05-17

**Authors:** Victoria Volodina, Peter Challenor

**Affiliations:** ^1^ The Alan Turing Institute, British Library, 96 Euston Road, London, NW1 2DB, UK; ^2^ College of Engineering, Mathematics and Physical Sciences, University of Exeter, Exeter EX4 4QE, UK

**Keywords:** emulation, Bayesian methods, error estimates

## Abstract

Many computer models possess high-dimensional input spaces and substantial computational time to produce a single model evaluation. Although such models are often ‘deterministic’, these models suffer from a wide range of uncertainties. We argue that uncertainty quantification is crucial for computer model validation and reproducibility. We present a statistical framework, termed history matching, for performing global parameter search by comparing model output to the observed data. We employ Gaussian process (GP) emulators to produce fast predictions about model behaviour at the arbitrary input parameter settings allowing output uncertainty distributions to be calculated. History matching identifies sets of input parameters that give rise to acceptable matches between observed data and model output given our representation of uncertainties. Modellers could proceed by simulating computer models’ outputs of interest at these identified parameter settings and producing a range of predictions. The variability in model results is crucial for inter-model comparison as well as model development. We illustrate the performance of emulation and history matching on a simple one-dimensional toy model and in application to a climate model.

This article is part of the theme issue ‘Reliability and reproducibility in computational science: implementing verification, validation and uncertainty quantification *in silico*’.

## 1. Introduction

A computer model (simulator) is a coded representation of a true process of interest. We treat a computer model as a mathematical function *f* that takes varying values of input parameters denoted by a vector $\text{x}=(x_{1},\ldots ,x_{p})\in R^{p}$, and returns output *f*(**x**). Owing to the vast improvements in the power of computers, these models, in combination with mathematical modelling and past physical data, are used in the analysis of complex physical systems and decision support [1]. For instance, Galform, a model of galaxy formation, is employed to study the behaviour of galaxies in the presence of dark matter [2]. Another example of powerful and complex models are the three-dimensional General circulation models (GCMs) of the atmosphere and ocean, numerical models based on the calculation of the budgets of mass, energy and momentum on a grid of columns on a sphere [3]. These models allow scientists to improve their understanding of the Earth system and to project the future climate state. In particular, simulations and projections produced by the world-leading climate centres’ GCMs are analysed and compared in the Coupled Model Intercomparison Project (CMIP) [4]. These model results and comparisons serve as the basis for climate research [5]. In the civil service, analytical models are used in many ways from appraising and evaluating the impact of policy options to planning the current strategy based on future forecasts [6,7]. These examples demonstrate the importance of assessing the model reliability and reproducibility.

Reproducibility may seem an odd concept in computer models. If we take the same deterministic computer code and run it again, we will get the same answer, given some small variation due to computer architecture, different compilers or at least preserve the global features in output behaviour [8]. But if we change one of the model inputs by a small amount, within its error bounds, so it is not a ‘real’ change, this will change the output. Similarly, if two laboratories use different models of the same process, the results tend to vary. These two phenomena raise a number of important questions about the interpretation and communication of model results. Such issues have featured heavily in press coverage of computer modelling of COVID-19 (see for example [9]).

In this paper, we argue that instead of reporting a single model estimate, i.e. result from a single simulation or a mean of ensemble of runs, the variability in model results should be acknowledged and reported. This variability, or error about a model estimate, represents how confident modellers are in the results produced by their model. To obtain these estimates, modellers need to identify and quantify different sources of uncertainty.

Uncertainty quantification in computer models is important for a number of reasons. Firstly, the analysis of physical processes based on computer models is riddled with uncertainty, which has to be addressed to perform ‘trustworthy’ model-based inference such as forecasting (predictions) [1]. Secondly, reporting and communicating uncertainties encountered in models is crucial in maintaining credibility in the model and modelling group (a team of climate scientists and modellers). For example, in climate science, models are constantly being improved and developed, therefore maintaining credibility in the future as model-based information improves is critical [10]. In cases where a model is used for decision support in civil services, the reputation of a single state department or even the whole government, depending on the scale of decision, could be on the line. In particular, commissioners of analysis are warned of the potential damage to credibility caused by overconfidence in their analysis [6].

In this paper, we adopt the subjective Bayesian approach to deal with uncertainties encountered in computer models. Goldstein [1] praises the ability of the subjective approach to translate complex uncertainty judgements provided by modellers into a mathematical formulation. The probabilities and conditional probabilities are employed to represent modellers’ uncertainty about the quantities of interest and observations about these quantities, respectively. For complex applications such as climate modelling, subjective Bayesian approach is the only way to perform model-based inference about the physical process of interest by combining limited data with expert knowledge. In cases where we have a very large experiment, objective Bayes characterized by ‘neutral’ knowledge prior distribution could be adopted. However, [1] still considers such analysis as the approximation to the full subjectivist analysis. Bayesian emulation and history matching are well-established techniques in the uncertainty analysis of computer models. In this paper, we are interested to showcase these approaches to perform model-based inference about the physical process of interest in the presence of uncertainty. In §2, we consider a general classification of different types of uncertainty presented by [11]. In §3a, we introduce the emulator, which is a statistical representation of a computer model behaviour [12–14]. In other words, a fast approximation to the full model, but one which includes an estimate of its own uncertainty. We proceed to combine emulators with statements of uncertainty about the discrepancy between the model and the physical process and about the measurement uncertainty associated with the observation to perform history matching in §3b. History matching is a global parameter search approach used to learn unknown input parameter values [2,15,16]. A model with fitted values could be used to predict the future behaviour of the system [11]. Section 4 demonstrates how these presented methods work for a climate model example. We finish off §5 with a general conclusion and discussion of the importance of these methods for model reproducibility.

## 2. Sources of uncertainty

In general, uncertainties can be categorized as either aleatory or epistemic [17]. *Aleatoric uncertainty* is associated with the internal variability in the system, and therefore cannot be reduced by conducting more experiments or collecting more data. It is natural to employ a probabilistic framework to model this type of uncertainty. The second type, *epistemic uncertainty*, arises from the deficiency in the model, caused by limited knowledge about the phenomena. Contrary to aleatoric uncertainty, there is a possibility to reduce this type of uncertainty by improving our knowledge about the system.

The process of identifying and classifying various sources of uncertainty is complex and model specific. For instance, in climate science, GCMs suffer from *initial condition uncertainty*, since the climate state generated by a model is sensitive to the changes in the initial state [10]. To analyse and represent this type of uncertainty, the projections (long-term predictions) from a climate model are computed from the ensemble of possible initial conditions [18]. Another example is in government where analysts and modellers identify a special type of uncertainty, *deep uncertainty*, which corresponds to all the events whose impacts on the policy outcome are not clear [6].

**Figure 1.**
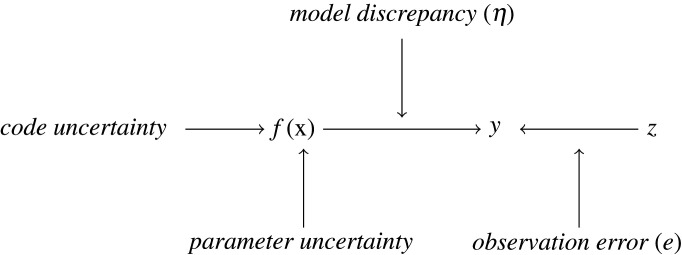
Schematic of the framework for analysing physical process *y* from computer model *f* and past physical data (observation) *z* and synthesizing all of the major uncertainties.

In this paper, we adopt the classification of sources of uncertainty provided by [11] and common to a wider class of models. Figure 1 depicts the main types of uncertainty encountered when dealing with computer model *f*(**x**), a representation of physical process *y*. *Parameter uncertainty* indicates that we have a number of unknown model parameters, whose values have to be estimated. These model parameters’ values could be learnt from the observation (physical data) of the process that the model describes, denoted by *z*. Calibration is the process of finding the input parameter values that allow the computer model to be a trustworthy representation of the physical process; and is commonly used to solve the inverse problem [11,19,20]. Since the actual observations of the physical process of interest are considered, the *observation error* (measurement uncertainty) *e* should be included as part of the estimation process.

Another source of uncertainty, named *model discrepancy*, denoted as *η*, arises from the notion that the model is not a perfect representation of the true process due to the process complexity and the lack of computational resources. The *model discrepancy* is a complex concept to grasp, and we provide an example with climate models. We previously mentioned that these models solve coupled PDEs discretized over the vertical and horizontal grids over the sphere numerically to study the effect of input variables of interest on the climate [4]. For GCMs, grids with cell sizes of the order of 100–300 km horizontally are typically used, which leads to the inability to calculate the effect of clouds explicitly [21]. These processes are referred to as ‘sub-grid scale processes’, and their effect is approximated inside the model. As a result, *model discrepancy*, the difference between the physical phenomena and the model representation, will appear.

To perform model-based inference and to learn about the relationships between parameters **x** and model outputs *f*(**x**), we are required to evaluate the model at many input parameter settings. In reality, *f* is computationally expensive to deal with, and therefore we treat *f* as uncertain at unseen **x**. *Code uncertainty* represents our uncertainty about *f*(**x**) at an arbitrary input value **x** [11]. An emulator conveniently provides a representation of the simulator behaviour together with the description of uncertainty about *f*(**x**) across the input space (see §3a for more details).

Apart from calibration, mentioned previously, there are other forms of analysis that a modeller might be interested in performing. Uncertainty analysis deals with the parameter uncertainty by looking at the distribution of the computer model output induced by a probability distribution on input parameters [22]. History matching, an efficient global parameter search approach, is another uncertainty analysis tool, considered in detail in §3b. It uses emulation to find input parameter space regions for which the simulator output agrees with the observation and includes our uncertainty judgements [23]. Sensitivity analysis looks at identifying how model input parameters affect the model outputs [24]. The main effect index (first-order Sobol’ index) and the total effect index (total Sobol’ index) are commonly used measures of sensitivity of computer model output to an individual parameter *x*_*i*_. The main effect index is based on considering the expected reduction in the uncertainty in the computer model output after we learn the true value of *x*_*i*_ [25]. The total effect index is based on quantifying the remaining uncertainty in the computer model output after we have learnt everything except *x*_*i*_ [26]. Sensitivity and uncertainty analysis are traditionally performed by employing a Monte Carlo algorithm. The algorithm proceeds as follows: the input parameter settings are drawn from the pre-defined probability distribution, the computer model runs are obtained at these parameter settings, which results in a random sample from the output distribution of interest. Kennedy & O’Hagan [11] pointed out that uncertainty and sensitivity analysis become impractical for expensive computer models and proposed to replace simulator with its surrogate. The emulator has been implemented as part of uncertainty and sensitivity analysis [22,24].

## 3. Methodologies in UQ

Here, we introduce a statistical model for the link between the computer model *f*(**x**) and physical process *y*, which contains major sources of uncertainty, described in §2. The main components of this framework are emulation and history matching. We employ these tools to derive the input parameter settings that result in acceptable matches between model output and observed data. We demonstrate the use and performance of each individual component on a simple one-dimensional toy model.

### (a) Emulation

In this paper, we consider a computer model as a black box model, i.e. the model is viewed in terms of its inputs **x** in *p*-dimensional parameter space X and outputs *f*(**x**). Most models are complex and require a significant computational time to produce a single run. Model simulation process could be a major bottleneck in model-based inference. Therefore, cheap approximation tools such as neural networks, splines and polynomial chaos could be used to approximate computer model behaviour across the input space [27–30]. Since we use an approximation, we should acknowledge and report our uncertainty about the computer model output (code). In this paper, we adopt a Gaussian process (GP) emulator as a surrogate to a complex computer model output. Contrary to other surrogates, GP emulator conveniently provides us with the measure of uncertainty about the generated prediction at an arbitrary input point **x**, often expressed as variances. This measure of uncertainty corresponds to the code uncertainty defined in §1.

We consider an emulator as a sum of two processes [8], defined as

3.1 $$\begin{array}{rl} & f(\text{x})=\sum_{j=1}^{q}\beta_{j}h_{j}(\text{x})+\epsilon (\text{x}),\\ & \epsilon (\text{x})∼GP\left(0,\sigma^{2}r(\cdot ,\cdot ;\delta )\right), \end{array}\}$$

where *h*(**x**) is a (*q* × 1) vector of specified regression functions in **x**, and **β** is a vector of corresponding regression coefficients to be fitted. The second component of the model, *ϵ*(**x**), is modelled as a zero-mean GP; the *r*( · , · ;**δ**) is pre-specified correlation function, and the **δ** is vector of its parameters (correlation lengths), *σ*^2^ corresponds to the variance parameter of the GP. We consider *h*(**x**)^*T*^**β** as a global response surface, capturing dominant features of computer model output and *ϵ*(**x**) as a correlated residual process, depicting local input dependent deviation from the global response surface. We can represent the output of a computer model by a GP and proceed to derive the GP prior for *f*(**x**) determined by a mean and covariance functions, i.e.

$$E[f(\text{x})]=h{\left(\text{x}\right)}^{T}\beta$$

and

$$\text{Cov}[f(\text{x}),f({\text{x}}^{\prime })]=\sigma^{2}r(\text{x},{\text{x}}^{\prime };\delta ).$$

Using probabilistic notation, we define the probability distribution for *f*(**x**) conditioned on the statistical model parameters {**β**, *σ*^2^, **δ**} as

3.2 $$f(\text{x})|\beta ,\sigma^{2},\delta ∼GP\left(h{\left(\text{x}\right)}^{T}\beta ,\sigma^{2}r(\cdot ,\cdot ;\delta )\right).$$

The regression functions in *h*(**x**) can be anything from simple monomials to Fourier transformations of **x** [31], based on the expert opinion on the simulator behaviour or regression modelling (stepwise regression) [32,33]. Williamson *et al.* [31] stated that *h*(**x**) could be used to add ‘physical insights’ into the statistical model. Meanwhile, the covariance function is used to characterize the similarity between the model output at two input points **x** and **x**′ and explicitly depends on the form of the correlation function. Williams & Rasmussen [34] provided the description of a number of commonly used correlation functions in the computer experiments literature. For instance, the power exponential correlation function is defined as

$$r(\text{x},{\text{x}}^{\prime };\delta )=\exp\left\{-\sum_{i=1}^{p}{\left(\frac{x_{i}-x_{i}^{\prime }}{\delta_{i}}\right)}^{\phi_{i}}\right\},$$

where *δ*_*i*_ > 0 and 0 < *ϕ*_*i*_ ≤ 2. In this paper examples, we use a squared exponential correlation function with *ϕ*_*i*_ = 2, *i* = 1, …, *p*. The components of **δ** are referred to as correlation lengths. We tend to obtain a stronger/weaker correlation for a pair of input points in the *i*th direction for larger/smaller values of the *i*th entry of the correlation length vector, i.e. *δ*_*i*_.

To complete the construction of GP emulators, we are required to obtain an ensemble of runs of the computer model for updating equation (3.2). Suppose we observe *n* computer model realizations F = (*f*(**x**_1_), …, *f*(**x**_*n*_)) at design points X = (**x**_1_, …, **x**_*n*_), and let the ensemble be denoted by {X, F}. By employing equation (3.2), the distribution of F is multivariate normal,

3.3 $$\text{F}|\beta ,\sigma^{2},\delta ∼MVN\left(H\beta ,\sigma^{2}K\right),$$

where *H* = [*h*(**x**_1_), …, *h*(**x**_*n*_)]^*T*^ is a (*q* × *n*) regression matrix, and *K* is (*n* × *n*) correlation matrix, with (*K*)_*ij*_ = *r*(**x**_*i*_, **x**_*j*_;**δ**). The result of updating is the posterior distribution *f*(**x**)|{X, F}, **β**, *σ*^2^, **δ**, i.e.

3.4 $$f(\text{x})|\left\{\text{X},\text{F}\right\},\beta ,\sigma^{2},\delta ∼GP\left(m^{*}(\text{x}),C^{*}(\cdot ,\cdot )\right),$$

with mean

$$m^{*}(\text{x})=E_{\text{F}}[f(\text{x})]=h{\left(\text{x}\right)}^{T}\beta +r(\text{x},\text{X})K^{-1}(\text{F}-H\beta )$$

and covariance

$$C^{*}(\text{x},{\text{x}}^{\prime })=Cov_{\text{F}}[f(\text{x}),f({\text{x}}^{\prime })]=\sigma^{2}[r(\text{x},{\text{x}}^{\prime })-r(\text{x},\text{X})K^{-1}r(\text{X},{\text{x}}^{\prime })],$$

where *r*(**x**, X) is *n*-vector whose *i*th component is *r*(**x**, **x**_*i*_), *i* = 1, …, *n*, the correlation between the point of interest **x** and the design point **x**_*i*_.

The GP model parameters are unknown and therefore have to be estimated or marginalized. [12] fixed values of GP hyperparameters at maximum likelihood estimates. Haylock & O’Haga [35] proposed to specify the non-informative prior for **β** and *σ*^2^, i.e. *π*(**β**, *σ*^2^) ∝ *σ*^−2^ and carry out the marginalization. The final step is to estimate the values of correlation length parameters **δ** either by finding the maximum likelihood estimate [11] or employing full Bayesian Markov chain Monte Caro (MCMC) methods. The probability distribution for *f* conditional on ensemble {X, F} and **δ** is Student process with *n* − *p* degrees of freedom. Oakley & O’Hagan [24] proposed to adopt a conjugate prior for **β** and *σ*^2^ in order to incorporate prior beliefs about simulator into the model-based inference. Full Bayesian MCMC methods were adopted by [8,20,36]. These approaches account for the uncertainty in GP hyperparameters but come at a higher computational cost.

In this paper, we obtain MAP (maximum *a posteriori*) estimates for GP hyperparameters by optimizing the posterior distribution function with subjective priors. To derive these values, we used ExeterUQ_MOGP [37], an R interface for fitting GP emulators and performing UQ, based on mogp_emulator, a Python package developed by the Research Engineering Group at the Alan Turing Institute [38].

**Figure 2.**
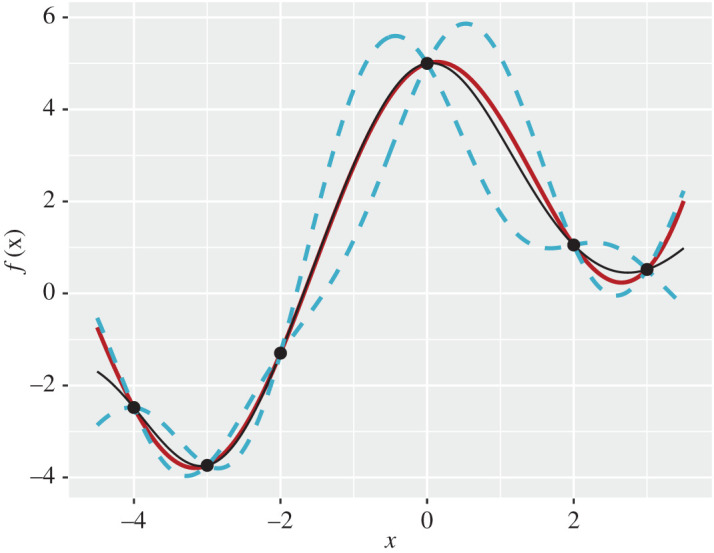
Plot of (true) function *f*(*x*), *x* ∈ [ − 4, 3] (red line). The black dots represent the observed data at 6 equally spaced values of *x*. The solid line represent the emulator’s updated expectation *E*_F_[*f*(**x**)], and the pair of blue dashed lines give the credible interval $E_{\text{F}}[f(\text{x})]\pm 2\sqrt{{\text{Var}}_{\text{F}}[f(\text{x})]}$, both as functions of *x*. (Online version in colour.)

We produced a GP emulator for a one-dimensional toy function. The true function has the following form:

f(x)=exp⁡(0.5x)+4cos⁡(x),

which has been computed at only 6 input points (training set X) evenly spread between *x*_1_ = −4 and *x*_6_ = 3. We specified the regression functions *h*(*x*) = (1, *x*, *x*^2^)^*T*^. In figure 2, the black solid and blue dashed lines correspond to *E*_F_[*f*(**x**)] and $E_{\text{F}}[f(\text{x})]\pm 2\sqrt{{\text{Var}}_{\text{F}}[f(\text{x})]}$. The true function *f*(*x*) is given by a solid red line, and it can be seen that it lies within the prediction interval for all *x*, except values close to the boundaries of input space. We observe that the point predictor goes through the six points of the training set and our uncertainty drops down to zero at these points. However, as we move farther away from these points, the length of prediction interval increases, indicating that our uncertainty about model behaviour grows.

We presented GP emulator for a deterministic model, which produces the same output under the same model conditions. However, stochastic models are being used increasingly in epidemic models [39], engineering [40] and climate and weather models with stochastic parameterization [41,42] . The outputs of stochastic simulators possess inherent randomness. In this case, the presented emulation approach could be extended by including an independent noise term *ν*( · ). The log variance, log (*ν*^2^( · )), is another GP [39,43].

### (b) Connecting model to reality

Emulation has two attractive features in our analysis. Firstly, emulators are extremely fast to evaluate, they can replace the original model in any larger calculation. Secondly, we consider the emulator as a probabilistic description about the value of the simulator at each input value, which we proceed to employ in the statistical model that establishes a relationship between observations of real system *z* and output of a model *f*. Kennedy & O’Hagan [11] introduced the ‘best input approach’ model that represents observation *z* via

z=y+e,

where *y* corresponds to the quantity of interest being observed (measured) and *e* corresponds to the error on this observation. These two quantities combined together give rise to the observation *z*. In this model specification, *z*, *y* and *e* represent random quantities. Observation error term *e* is assumed to be independent from *y*, and unbiased with zero mean and variance Var[*e*].

The ‘best input approach’ assumes the existence of a ‘best input’ setting x* so that computer model represent the physical process within a specified statistical model

$$y=f({\text{x}}^{*})+\eta ,$$

where *η* is a model discrepancy term (structural error). This term indicates that model simulated at its ‘best input’, *f*(x*), would still not be in agreement with the real physical system *y*. In particular, model discrepancy allows modellers to include any extra information about model’s deficiencies in their model of real physical process [23]. We consider *y*, *f*, x* and *η* as random quantities and specify zero expectation and variance Var[*η*] for *η*. The model discrepancy random quantity could be estimated based on expert judgements in combination with simulations from a more complex computer model [31].

By employing a statistical model described above together with emulation, we could perform a global parameter search. History matching attempts to find input parameter values to achieve the consistency between observations and computer model representation. The goal of history matching is to identify the regions of input space corresponding to acceptable matches, and this is performed by ruling out the implausible regions iteratively in waves. In particular, we are trying to rule out regions in X that could not contain x* given the uncertainty specification and using the implausibility function, that has the following form:

3.5 $$I(\text{x})=\frac{|z-E_{\text{F}}[f(\text{x})]|}{\sqrt{{\text{Var}}_{\text{F}}[z-E_{\text{F}}[f(\text{x})]]}}.$$

We proceed to rewrite the denominator of the implausibility function as

$${\text{Var}}_{\text{F}}[z-E_{\text{F}}[f(\text{x})]]=\text{Var}[e]+\text{Var}[\eta ]+{\text{Var}}_{\text{F}}[f(\text{x})].$$

To perform history matching, we require an emulator, so that we can obtain expectation, *E*_F_[*f*(**x**)], and variance, Var_F_[*f*(**x**)], for any input setting **x**. For model output *f* and observation *z*, large values of I(x) at any **x** imply that, relative to our uncertainty, the predicted output of computer model at **x** is very far from where we would expect it to be if *f*(**x**) were consistent with *z*. However, small values of implausibility function imply either that we expect the model to be close to our observations for **x** or that we are very uncertain about the model behaviour, i.e. Var_F_[*f*(**x**)] is large. We are required to specify a value of a threshold, *a*, so that **x** at which I(x)>a is deemed as implausible. For instance, [44] proposed to set the value of *a* to be 3 following 3 sigma rule. The remaining parameter space is termed as Not Ruled Out Yet (NROY) and defined as [16]

$$X_{1}=X_{NROY}=\{\text{x}\in X:I(\text{x})\leq a\}.$$

We could perform history matching iteratively. Refocussing is the process of performing history matching multiple times, by deriving wave *k* NROY space from the parameter space $X_{k-1}$. We start by defining NROY space in wave *k* as

$$X_{k}=\left\{\text{x}\in X_{k-1}:I(\text{x},{\text{F}}_{\left[k\right]}\leq a)\right\}.$$

We compute $I(\text{x};{\text{F}}_{\left[k\right]})$ across the $X_{k-1}$ using expectation and variance produced by an emulator for *f*(**x**) defined inside $X_{k-1}$. To construct this emulator, we are required to obtain the design

$${\text{X}}_{\left[k\right]}={\left({\text{x}}_{k,1},\ldots ,{\text{x}}_{k,n_{k}}\right)}^{T}\in X_{k-1},$$

together with the corresponding computer model simulations, i.e.

$${\text{F}}_{\left[k\right]}=(f({\text{x}}_{k,1}),\ldots ,f{\left({\text{x}}_{k,n_{k}})\right)}^{T}.$$

We retain **x** as part of wave *k* NROY space only if it has not been ruled out (RO) in the previous *k* − 1 waves of history matching.

Refocussing is considered as a powerful method and has been applied across the wide range of fields including galaxy formation [2,45], HIV transmission model [46,47] and climate [31,48]. At each iteration (wave), we increase the density of ensemble, which leads to the improvement in the performance of statistical emulators, i.e. more accurate predictions and lower uncertainty about the predictions. Therefore, we expect to retain as part of NROY space input parameters setting at which model output is close to the observation.

**Figure 3.**
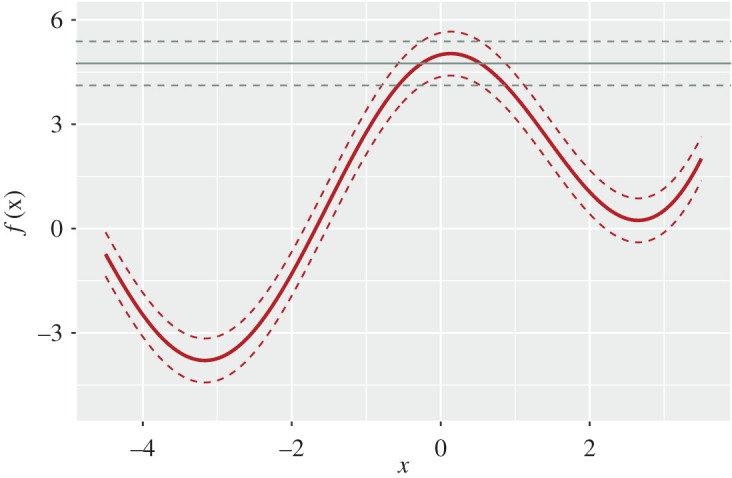
The model *f*(*x*) is given by the red line, the observed data *z* by the horizontal grey line. We include both observation error *e* (the grey dashed lines represent $z\pm 2\sqrt{\mathrm{Var}[e]}$) and model discrepancy *η* (the red dashed lines show $f(\text{x})\pm 2\sqrt{\mathrm{Var}[\eta ]}$). (Online version in colour.)

We perform history matching on a simple one-dimensional toy model introduced in §3a. Figure 3 depicts the one-dimensional toy model with both observation error *e* and model discrepancy *η*. Here, we show the model behaviour *f*(*x*), given by the red solid line, together with model error (the red dashed lines represent $f(x)\pm 2\sqrt{\text{Var}[\eta ]}$). The observation *z* is given by the solid grey line together with the observation error (the grey dashed lines show $z\pm 2\sqrt{\text{Var}[e]}$). We specify the following values for our demonstration: *z* = 4.75, Var[*e*] = 0.1 and Var[*η*] = 0.1

**Figure 4.**
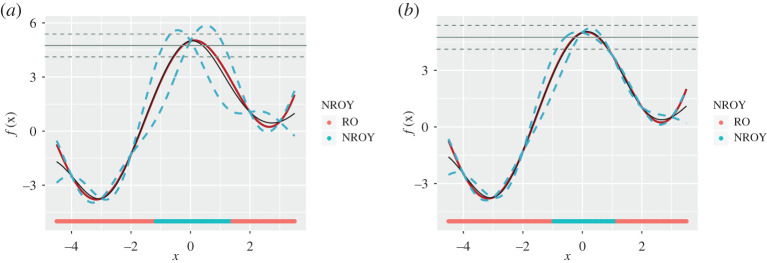
(*a*) The emulator expectation and credible intervals as in figure 2; however, now the observation *z* plus observed error has been included as the horizontal grey solid and dashed lines respectively. The implausibilities I(x) are represented by the colours on the *x*-axis: red and green for high (I(x)>3) and low (I(x)<3) implausibility respectively, with the green interval defining the non-implausible region $X_{1}$. (*b*) The second wave is performed by evaluating an additional point located within $X_{1}$. The emulator becomes more accurate over $X_{1}$ and the implausibility more strict, hence defining the smaller non-implausible region $X_{2}$, given by the green interval. (Online version in colour.)

In figure 4*a*, we demonstrate the emulator expectation and credible intervals as in figure 2 together with the observation plus observed error. The colours on the *x*-axis correspond to the implausibility values I(x): red and green for RO (I(x)>3) and NROY (I(x)<3), respectively.

The function *f*(*x*) is defined on −4.5 < *x* < 3.5. We proceed to perform history matching and specify the threshold *a* at 3 to obtain the wave 1 NROY space $X_{1}$, shown by the green region on the *x*-axis in figure 4*a*, i.e. −1.16 < *x* < 1.37. We perform the second wave of history matching by obtaining an additional model run within the $X_{1}$ space, reconstructing the emulator, and recalculating the implausibility measure I(x). In figure 4*b*, we observe that the emulator has become more accurate together with reduced uncertainty about its predictions. As a result, the non-implausible region in green has shrunk, i.e. −0.96 < *x* < 1.13. In case if we ignored observation error, model discrepancy and code uncertainty in our analysis, only two values of *x* can be viewed as acceptable while all others are unacceptable.

## 4. Example

We proceed to demonstrate the use of emulation and history matching in the climate model context. In particular, this example is inspired by the work that has been done as part of the ANR (Agence Nationale de la Recherche) funded HIGH-TUNE project. The main objective of HIGH-TUNE project is to improve the representation of the boundary-layer clouds in two French General Circulation Models, ARPEGE-Climat and LMDZ. These types of clouds play a crucial role in the water and energy cycles of the atmosphere and impact surface temperature at various scales [49]. However, boundary layer clouds are much smaller than a grid cell of a climate model, and therefore the collective behaviour and the effect on the large-scale model outputs of an ensemble of boundary-layer clouds is parameterized. The parameterization schemes depend on a variety of ‘free’ parameters and calibrating (tuning) these parameters is crucial to avoid biases in the global climate models [4].

In particular, climate modellers are interested in finding the regions of input parameters, that correspond to acceptable matches between single-column simulations of the GCMs (SCMs) and three-dimensional high-resolution large eddy simulations (LESs). Note that LES runs are treated as surrogate observations since it is challenging to collect data on clouds’ properties at the required temporal and spatial scale [50]. In climate modelling, this type of tuning is termed ‘process-based tuning’.

A number of approaches could be used to perform climate model tuning. The uncertain parameters could be adjusted manually, which is referred to as expert tuning [51–54]. However, this approach suffers from a lack of objectivity as well as being very time-consuming and often requires thousands of runs of a climate model. Another approach is based on specifying and optimizing a cost function with respect to calibration parameters that measures the distance between model simulations and a collection of observations [55,56]. However, constantly evaluating climate models at new parameter settings is time-consuming, especially as the complexity of the model increases. The solution to this problem could be to construct surrogate models (emulators) for target metrics and use surrogate models’ outputs in the cost function computation [51,57]. However, this framework fails to account for a number of important sources of uncertainty.

We proceed to present the application of Bayesian emulation and history matching to climate model tuning. For demonstration, we consider the statistics on water vapour at 500 m (qv500) generated by the SCM by varying five input parameters, that are part of convection parameterization, for two cases SANDU [58] and ARMCU [59]. These two cases correspond to the specific characteristics of the physical representation of boundary-layer clouds. This metric for two cases has been averaged over a few hours, for more details see [60][Table 2]. The experiment aimed to derive the acceptable matches between SCM’s output and LES for these two cases, and we treated them as two independent outputs, i.e. *f*_*i*_(**x**), *i* = 1, 2.

First, we produced a space-filling design X over the whole input space X, i.e. a 90-point maximin Latin hypercube design [61]. We evaluated model at the obtained design for each case. To construct a GP emulator, we were required to specify the form of the regression function, *h*(**x**), and the prior distributions for GP parameters, i.e. **β**, **δ**, *σ*^2^. We obtained the form of a mean function by using a stepwise regression procedure [62]. We used a forwards and backwards stepwise selection routine and considered interaction terms, higher-order polynomials together with the Fourier functions for selection. Similar procedure has been used by [33]. We used the default prior specification setting implemented as part of Exeter_MOGP [37]. In particular, we specified a uniform prior for intercept and N(0, 10^2^) for the regression coefficients. These weakly informative priors rule out unreasonable parameter values but are not so strong to rule out values that might make sense in the context of model data. A logNormal(0, 0.125) was defined for correlation length parameter. We specified a subjective prior for the variance parameter *σ*^2^, so that together with the prior specifications for **δ** the confounding between *σ*^2^, regression line and **δ** could be resolved. Finally, MAP estimates were obtained for GP hyperparameters.

**Figure 5.**
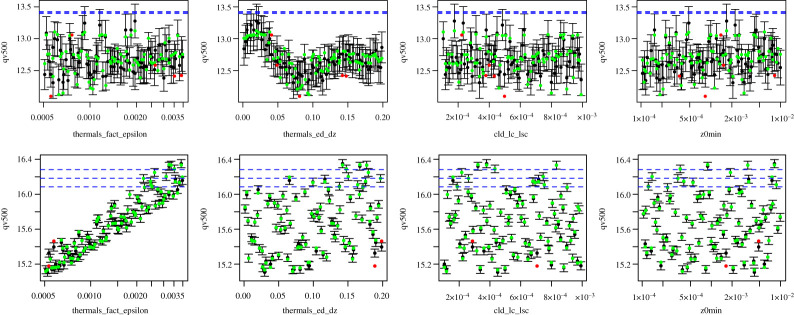
Leave-One-Out diagnostics plots against each of the parameters for SANDU (top row) and ARMCU (second row) cases on original input scales. The predictions and two standard deviation prediction intervals are in black. The true model values are in green if they lie within two standard deviation prediction intervals, or red otherwise. The observation *z* plus observed error ($z\pm 2\sqrt{\text{Var}[e]}$) are shown by blue dashed lines. (Online version in colour.)

Prior to history matching, we ran diagnostic checks to assess the performance of the obtained emulators. In particular, Leave-One-Out cross-validation was performed, where each point from the design set was retained for validation, while the emulator was refitted. Figure 5 shows the diagnostic plots for four of the model parameters for SANDU (top row) and ARMCU (bottom row) cases. Black points and error bars (±2 s.d. prediction intervals) are computed from *E*_F_[*f*(**x**)] and *Var*_F_[*f*(**x**)]. The true (left out) values are plotted in green/red if they lie within/outside two standard deviation prediction intervals.

The plots in the second row indicate that the emulator represents model well for ARMCU case with small error bars and predictions close to true values. In the top row of figure 5, we observe four points outside the uncertainty bounds. Since 2 standard deviations correspond to 95% confidence intervals, we expect 5% of points to be red and outside the error bars. We also obtain larger error bars due to more sophisticated behaviour of model for SANDU case. Overall, we are happy with the emulators’ representation of the model outputs and proceed to perform history matching.

We provide the specification of *z*, Var[*e*] and Var[*η*] for each case in table 1. We imposed the implausibility constraint simultaneously across two outputs, by employing $I_{M}<c$ with conservative cutoff *c* of 3, where $I_{M}(\text{x})$ is the maximum implausibility measure defined by $I_{M}(\text{x})=\max_{i}I_{i}(\text{x})$, *i* = 1, 2. The alternative measure is to consider the second implausibility, which allows for some inaccuracy of the emulators. We performed a single wave of history matching and obtained $X_{1}$, the NROY space remaining after wave 1, which has a size of 28% of the original input space X.

**Table 1.** Summary information for history matching.

case	observation *z*	observation error Var[*e*]	model discrepancy Var[*η*]
SANDU	13.41	0.0078	0.040
ARMCU	16.18	0.049	0.001

**Figure 6.**
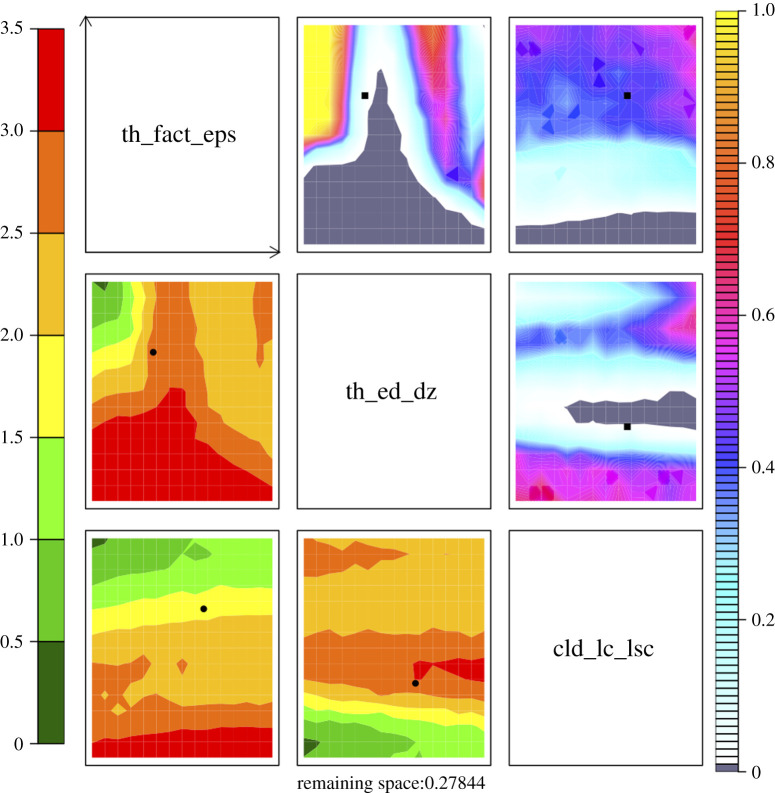
NROY density plots (upper triangle) and minimum implausibility plots (lower triangle). Each panel plots either NROY density or minimum implausibility for a pair of parameters. NROY densities, for each pixel on any panel in the upper triangle, represent the proportion of points in the input space behind that pixel that are NROY and are indicated by the colour whose scale is indicated on the right. Grey coloured regions are completely ruled out. Minimum implausibilities, for each pixel on any panel on the lower triangle of the picture, represent the smallest implausibilities found in input space. These plots are oriented the same way as those on the upper triangle, for the ease of visual comparison. Currently used parameter values in GCM is depicted as the square on the NROY density plots and as the circular point on the minimum implausibility plots. (Online version in colour.)

We proceeded to investigate two-dimensional representations of the shape of non-implausible region $X_{1}$ for a selection of input parameters. The NROY density and minimum implausibility plots are shown in figure 6. These plots are produced by computing the implausibility function, given in equation (3.5), at a large number of points within the five-dimensional input space. Each panel on the upper triangle corresponds to the density of points in the NROY space. Grey regions are completely ruled out, which indicates that for a fixed value of two parameters under consideration, we are unable to retain any parameter settings in the other three dimensions. Each panel on the lower triangle shows the minimum implausibility plot. The value behind each pixel corresponds to the smallest implausibility found at the fixed value of a pair of parameters. The red regions indicate high implausibility, and we do not expect to observe good matches between climate model and data in these regions of the input space. Green/yellow regions correspond to the low implausibility values and the location of potentially ‘good’ input parameters settings. In general, we are interested in investigating further these regions in the subsequent waves of history matching.

Figure 6 provides modellers with insights into the relationships between a pair of input parameters. In particular, we observe that there is a non-linear relationship between parameters thermals_fact_epsilon and thermals_ed_dz, i.e. these parameters should be varied jointly to obtain NROY models. There is a very limited effect from cld_lc_lsc in our analysis. By performing a single wave of history matching, we managed to rule out more than 70% of input space. We note that the default values for the selected parameters shown in figure 6 lie inside the NROY space. These values were obtained by performing a slow expert tuning [60]. The second wave of history matching could be performed starting with obtaining climate model runs in the green/yellow regions and updating GP emulators.

## 5. Discussion

Increasingly science (and policy) is relying on numerical models of the physical world. The credibility of such models depends in part on the reproducibility of computer experiments. A large part of reproducibility comes from having well maintained and open code [63]. In this paper, we argue that reproducibility in computer models is not just about having the correct code, important though that is, it is about the relationship between the inputs and outputs of the model as well as its relationship to data from the real world. Any results need to be presented with uncertainty bounds of some form and all assumptions (priors, etc.) need to be clearly and openly stated.

We have presented approaches that derive regions in input space at which we expect to achieve consistency between the computer model and observations of the system. In particular, the use of emulators and history matching allows modellers to explicitly include major sources of uncertainty discussed in §2. Simulating model at the obtained input settings results in a range of predictions, that we argue should be reported and analysed to ensure the reproducibility criteria are met.

We believe that the presented statistical analysis is important for model development. Brynjarsdóttir & O’Hagan [64] pointed out that modelling is an iterative process, i.e. by comparing the model to the observed data modellers could learn about model deficiencies to work on for the next release. Stainforth *et al.* [10] argued that uncertainty assessment and model development are part of the ‘two-pronged approach’.

We recognize that the process of identifying sources of uncertainty is subjective and model dependent. For instance, there is still ongoing research in formulating model discrepancy, since the poor representation of this term leads to biased and over-confident input parameter values that cannot be used to generate trustworthy model predictions [64]. To specify model discrepancy Var[*η*], modellers’ knowledge and opinions could be extracted via prior elicitation [65] in combination with simulations produced by a high-resolution model [10,31].

We need to mention an alternative probabilistic approach to history matching, a Bayesian calibration. Kennedy & O’Hagan [11] obtained a posterior distribution for input parameters of interest (calibration parameters), i.e. *π*(x*|{X, F, *z*}), which takes into account major sources of uncertainty. However, we choose to employ history matching in our study of complex computer models because of its flexibility to specify the different form of implausibility functions as well as the set of metrics of interest [2,48]. Another reason is that the result of Bayesian calibration is always the probability distribution for x* over the input space X. If the computer model cannot represent the system, then the calibration distribution is unfit to be employed in the further analysis. By contrast, history matching would rule out the entire input parameter space, which indicates that the computer model is unacceptable to represent the true physical system.

In this paper, we performed an analysis based on a single model. However, in climate science, it is common to perform inference about the physical process based on multiple models, i.e. ‘ensemble’ of different simulators or ‘multi-model ensemble (MME)’. For this type of problem, [66] proposed to take into account the shared discrepancies among the simulators. In particular, the statistical model (mimic) is specified to represent the common structure in the behaviour of simulators. Therefore, these simulators are characterized by the parameters of a statistical model (descriptor). To model the relationship between the simulator and the real climate, [66] assumed that the simulator descriptor is centred upon *θ*_0_ + *ω*, where *θ*_0_ is a real climate descriptor and *ω* is an ensemble-specific discrepancy.

## Data accessibility

This article has no additional data.
